# Alcohol consumption, mental health, and the moderating role of social isolation during the COVID-19 pandemic in southern Brazil

**DOI:** 10.1590/1980-549720260002

**Published:** 2026-01-30

**Authors:** Francine Costa, Thais Martins-Silva, André Luiz Girotto, Laura Cunha Goulart, Victor Alves Modesto e Silva, Rafaela Costa Martins, Cauane Blumenberg, Marina Xavier Carpena, Juraci Almeida Cesar, Christian Loret de Mola

**Affiliations:** I Universidade Federal do Rio Grande – Rio Grande (RS), Brazil.; II Universidade Federal de Pelotas – Pelotas (RS), Brazil.; III Universidad Cientifica del Sur – Lima, Peru.

**Keywords:** Maternal mental health, Alcohol consumption, COVID-19 pandemic, Social isolation, Saúde mental materna, Consumo de álcool, Pandemia de COVID-19, Isolamento social

## Abstract

**Objective:**

Investigate changes in alcohol consumption and the association between maternal depression and anxiety, considering the moderating effect of social isolation during the COVID-19 pandemic, using a population-based cohort from Brazil.

**Methods:**

Data were obtained from the WebCovid-19 study, a web-based follow-up of the 2019 Rio Grande (RS), Brazil, birth cohort, with 1,077 and 1,033 postpartum women participating in waves I and II, respectively. Changes in maternal alcohol consumption were self-reported, while depression and anxiety were assessed using the Edinburgh Postnatal Depression Scale and the Generalised Anxiety Scale. Crude and adjusted negative binomial regression was conducted, including tests for moderation by social isolation.

**Results:**

Of the 781 mothers included, 57.3% reported staying home ≥5 days in the last week, and 5.0% increased alcohol consumption during the pandemic. Median depression and anxiety scores were 8.0 (interquartile range — IQR 3–13) and 6.0 (IQR 3–10), respectively. Mothers who increased alcohol consumption had a 5-point (95% confidence interval — 95%CI 3.0–7.0) and 4.2-point (95%CI 2.6–5.9) increase in depression and anxiety scores, respectively. Social isolation duration did not significantly modify the effect of alcohol consumption on mental health.

**Conclusion:**

Increased alcohol consumption during the pandemic was associated with higher depression and anxiety scores. The hypothesised moderating effect of longer isolation on this association remains a possibility.

## RESUMO

**Objetivo:**

Investigar as mudanças no consumo de álcool e a associação com depressão e ansiedade materna, considerando o efeito moderador do isolamento social durante a pandemia de COVID-19, utilizando uma coorte populacional do Brasil.

**Métodos:**

Os dados foram obtidos do estudo WebCovid-19, um acompanhamento baseado na web da coorte de nascimentos de 2019 do Rio Grande (RS), Brasil, com 1.077 e 1.033 mães participantes nas ondas I e II, respectivamente. As mudanças no consumo de álcool materno foram autorrelatadas, enquanto depressão e ansiedade foram avaliadas por meio da Escala de Depressão Pós-Natal de Edimburgo e da Escala de Ansiedade Generalizada. Foram realizadas regressões binomiais negativas brutas e ajustadas, incluindo testes para moderação pelo isolamento social.

**Resultados:**

Das 781 mães incluídas, 57,3% relataram ficar em casa ≥5 dias na última semana, e 5,0% aumentaram o consumo de álcool durante a pandemia. As medianas dos escores de depressão e ansiedade foram 8,0 (IQR 3–13) e 6,0 (IQR 3–10), respectivamente. Mães que aumentaram o consumo de álcool apresentaram aumento de 5 pontos (IC95% 3,0–7,0) e 4,2 pontos (IC95% 2,6–5,9) nos escores de depressão e ansiedade, respectivamente. A duração do isolamento social não modificou significativamente o efeito do consumo de álcool na saúde mental.

**Conclusão:**

O aumento do consumo de álcool durante a pandemia foi associado a escores mais elevados de depressão e ansiedade. O efeito moderador hipotetizado do isolamento prolongado nessa associação permanece uma possibilidade.

## INTRODUCTION

In March 2020, the World Health Organization (WHO) declared the SARS-CoV-2 pandemic, marking a significant moment in global health history due to its rapid and widespread geographic reach^1^. This pandemic had an important impact on mental health issues, with a significant increase in depressive symptoms and other emotional disturbances^2,3^. Mothers in the postpartum period comprise an especially vulnerable group, as this period is a risk factor for mental health problems^4^. For instance, 20% of mothers of live newborns from high-income countries (HIC) are expected to develop depression in the first three months after delivery^5^. In Brazil, a recent study found that the prevalence of post-partum depression was 25% among mothers who were beneficiaries of the cash transfer program^6^.

Previous evidence indicates that mental health problems, such as anxiety and depression, are frequently correlated with substance abuse, including alcohol. However, the mechanisms involved in this association are still unclear^7-9^. Current evidence highlights that the association between alcohol consumption and depression can be bidirectional^10,11^. Long-term abstainers and heavy alcohol users are more likely to have depressive episodes when compared to moderate users^12^. Moreover, it is suggested that social and cultural contexts may explain the variation of this association^13^. Stressful circumstances, such as the pandemic, also had the potential to affect mental health and stress responses. Also, pandemic-related stress can increase as social isolation increases, leading to maladaptive coping behaviours, such as alcohol consumption, which can become highly addictive and disrupt normal activities^14,15^.

Studies have indicated an increase in the harmful use of alcohol during periods of social isolation associated with the pandemic, leading to a rise in the prevalence of various adverse outcomes, including harmful drinking, withdrawal symptoms, intimate partner violence, harm to children, suicide, mental health issues, and non-communicable diseases^14^. A cross-sectional survey identified that 13.8% of adults worldwide who consumed alcohol before the pandemic reported an increase in episodes of heavy drinking during the pandemic^15^. Additionally, a recent study involving Australian parents of children and adolescents aged zero to 18 years found higher rates of parental depression, anxiety, stress, and increased alcohol consumption during the pandemic compared to pre-pandemic levels^16^. Consequently, mothers with alcohol abuse issues tend to have poorer relationships with their children^17^).

The impact of social isolation measures on mental health outcomes is not well understood. This study aimed to analyse the effect of increased alcohol consumption during the COVID-19 pandemic on the mental health of mothers from the 2019 Rio Grande birth cohort in Brazil. Additionally, it examined whether social isolation might modify this association. We hypothesised that increased alcohol consumption during the pandemic would elevate the risk of depression and anxiety, and that mothers who experienced prolonged social isolation would be at an even greater risk for these mental health issues.

## METHODS

The Rio Grande Birth Cohort began in 2019, when all hospital births occurring in Rio Grande, Southern Brazil, were identified. Rio Grande is a mid-sized coastal city with a population of approximately 212,673 residents, ranked 698^th^ in gross domestic product (GDP) in 2020. It is served by two leading hospitals that handle all hospital births in the municipality. The city records approximately 2,500 live births per year, providing a stable framework for population-based birth cohort studies^18,19^. We invited mothers who gave birth to newborns weighing ≥500 g or at least 20 weeks of gestational age to respond to a standardised face-to-face questionnaire. Out of 2,365 eligible mothers, 2,313 agreed to participate (response rate of 97.8%). The birth cohort's follow-up studies were conducted online due to the pandemic and were named WebCovid-19. Mothers were eligible for the WebCovid-19 study if they had given birth to a single child at the baseline study and lived in an urban area of the city (n=2,051). Between May 11 and July 20, 2020, all eligible mothers were invited to participate in the first wave of WebCovid-19 (Wave I) through a web-based questionnaire. Wave II followed from August to December 2020. Response rates were 54.1 and 50.7% for Waves I and II, respectively. More information can be found in Martins et al.^19^

For the current study, we employed a cross-sectional approach, using data from Wave II only. Mothers were invited to participate in the survey through social media, WhatsApp, or telephone contact by trained personnel. A link was sent to mothers who agreed to participate in the study. If the mother preferred to be interviewed or had difficulties answering the online form, the questionnaire was administered by telephone. The development of the survey and data collection was conducted in the REDCap software^20^.

### Outcomes

We used the Edinburgh Postnatal Depression Scale (EPDS) and the Generalised Anxiety Scale (GAD-7) to evaluate depressive and anxiety symptoms, respectively. The EPDS is a 10-item scale, ranging from 0 to 30 points, developed in 1987 by Cox et al.^21^ for screening postpartum depression, whose use has been validated in Brazilian populations^22^. The GAD-7 is a 7-item scale, scoring from 0 to 21 points, for screening and rating the severity of generalised anxiety disorder (GAD). The GAD-7 was also validated for application in Portuguese^23^. For the present study, both EPDS and GAD-7 were analysed as numeric variables.

### Exposure

We asked mothers about the impact of the pandemic on the amount of alcohol consumed. Participants were directly asked about their alcohol consumption since the beginning of the pandemic through the following question: "Since the beginning of the pandemic, has your alcoholic drinking consumption changed?".

The answer options were:

- I do not drink;
- Yes, it has increased;
- Yes, it has decreased;
- No, it has remained the same.

For the current study, the exposure was categorised as "never drank", "decreased", "not changed", or "increased" alcohol consumption.

### Effect modifier

Social isolation was evaluated as an effect modifier. For that, we asked, "How many days did you leave home in the previous week?", with answer options ranging from none to seven days. For the current study, the answers were categorised as "up to two days", "three to four days", and "five days or more".

### Confounding variables

The following variables collected from the baseline were considered confounders, based on prior evidence of their associations with both alcohol use and mental health outcomes: self-reported skin colour (black, brown, white), maternal education (elementary school, high school, college, or more), number of people residing in the household (<3, 3 or 4, 5 or more) and family income (in Brazilian *Reais* — BRL — and classified in tertiles: 1^st^ as poorest and 3^rd^ as richest). Skin colour, maternal education, and family income were included as markers of social inequality and socioeconomic status. At the same time, household size, as a proxy for potential crowding, could influence the degree of social isolation experienced during the pandemic. The selection of confounders was guided by Directed Acyclic Graphs (DAGs), when applicable, or by prior literature and theoretical knowledge.

### Data analysis

Descriptive analysis of the absolute and relative frequencies of the variables was performed. Given that the outcome variables (scores of depressive and anxiety symptoms) were count-based and presented over dispersion (i.e., variance greater than the mean), the association between alcohol consumption and the scores of depression and anxiety symptoms was investigated using crude and adjusted negative binomial regression. This approach is recommended when standard Poisson models may underestimate standard errors and overstate statistical significance^24,25^.

The results were shown as the median and interquartile range (IQR), incidence rate ratio (IRR), and their respective 95% confidence intervals (95%CIs), indicating the relative change in symptom counts associated with exposure. Although the IRR is traditionally used for event-based data, it is also appropriate in cross-sectional analyses of symptom scores modelled as count variables. No threshold was applied to dichotomise the outcomes, as the aim was to assess symptom severity across a continuous scale rather than classify clinical diagnoses.

Interaction analyses were performed using days of social isolation as an effect modifier. Given the typically low statistical power of interaction tests in epidemiological studies, we adopted a more liberal significance threshold (p<0.20) to reduce the likelihood of overlooking potentially meaningful effect modification^26^. Only mothers with complete data on all items of the depression and generalised anxiety disorder questionnaires were included in the analyses. All statistical analyses were conducted using Stata 16.0 (StataCorp, College Station, TX).

### Ethical aspects

All participants signed an informed consent form based on the ethical principles of the Declaration of Helsinki. The research was approved by the ethics committee of the Federal University of Rio Grande (protocol number 15724819.6.0000.5324).

### Data Availability Statement

The entire dataset supporting the findings of this study is available upon request to the corresponding author.

## RESULTS

**Figure 1:**
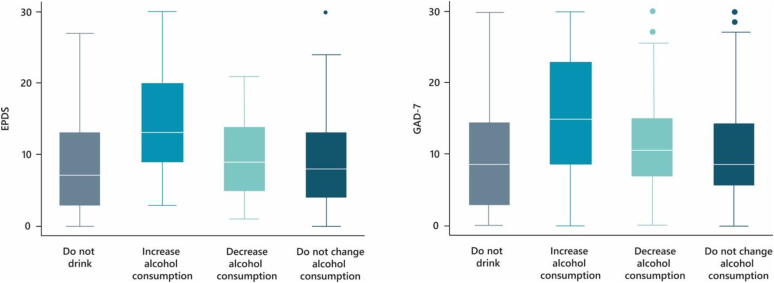
Description of depression (Edinburgh Postnatal Depression Scale — EPDS) and anxiety scores (Generalized Anxiety Scale — GAD-7) in the sample, according to alcohol consumption. 2019 Rio Grande (RS), Brazil, Birth Cohort.

During the WebCOVID-19 wave II, the mean age of the children was 13.6 months (standard deviation [SD] 3.7 months). Out of 1,040 respondent mothers in this wave, 781 had complete data for depression and anxiety and were included in the analysis. Most declared themselves to be white (80.0%), had completed high school or less (47.4%), lived in a household with three or more residents (33.4%), and belonged to the richest tertile of family income (42.2%). Regarding social isolation, most mothers reported staying at home for ≥5 days in the last week (57.3%). An increase in alcohol consumption during the pandemic was reported by 5.0% of the mothers, while for 16.0% it did not change, and for 5.6% there was a decrease in alcohol consumption. The median scores of depression and anxiety were 8.0 (IQR 3–13) and 6.0 (IQR 3–10), respectively (Table 1). Figure 1 shows mental health scores according to categories of alcohol consumption. Both depression and anxiety median scores were higher for mothers who increased their alcohol consumption during the pandemic (Figure 1). Comparison with non-included participants reveals that the analytic sample was relatively advantaged: the mothers included were more likely to be white, have higher levels of education, belong to the richest income tertile, reside in smaller households, and spend more days at home during the pandemic. Alcohol consumption patterns were similar between groups. These differences suggest that mental health symptom prevalence may be underestimated, and associations between alcohol use, time at home, and mental health outcomes may not fully reflect more socioeconomically vulnerable mothers (Supplementary Table 1).

**Table 1:** Sample description of baseline and WebCovid-19 wave-II. 2019 Rio Grande (RS), Brazil, Birth Cohort.

Variable		Baseline (n=2,051)		WebCovid-19 wave II(n=781)	
		n	95%CI	n	95%CI
Maternal self-reported skin color					
	Black	174	8.5 (7.3–9.8)	49	6.3 (4.8–8.2)
	Brown	302	14.7 (13.2–16.3)	107	13.7 (11.4–16.3)
	White	1,576	76.8 (74.9–78.6)	625	80.0 (77.1–82.7)
Maternal schooling					
	Elementary school	607	29.6 (27.7–31.6)	163	20.9 (18.1–23.9)
	High school	978	47.6 (45.5–49.8)	370	47.4 (43.9–50.9)
	College or more	467	22.8 (21.0–24.6)	248	31.7 (28.6–35.1)
Number of people in the household					
	<3	1,262	61.5 (59.3–63.6)	520	66.6 (63.2–69.8)
	3 or 4	422	20.6 (18.9–22.4)	147	18.8 (16.2–21.7)
	5 or more	368	17.9 (16.3–19.7)	114	14.6 (12.3–17.3)
Family income					
	1^st^ (poorest)	716	35.8 (33.7–37.9)	219	28.6 (25.5–31.9)
	2^nd^	597	29.8 (27.8–31.8)	223	29.1 (26.0–32.5)
	3^rd^ (richest)	690	34.4 (32.4–36.6)	323	42.2 (38.7–45.8)
Days in a week spent at home during the pandemic					
	5 days or more	-	-	447	57.3 (53.8–60.7)
	4 days or less	-	-	333	42.7 (39.3–46.2)
Alcohol consumption during the pandemic					
	Never drank	-	-	572	73.3 (70.1–76.3)
	Decreased	-	-	44	5.6 (4.2–7.5)
	Not changed	-	-	125	16.0 (13.6–18.8)
	Increased	-	-	39	5.0 (3.7–6.8)
		**n**	**Median (IQR)**	**n**	**Median (IQR)**
Depression score		2020	18.4 (2.6)	781	8.0 (3–13)
Anxiety score		2045	6.2 (4.0)	781	7.1 (5.6)

95%CI: 95% confidence interval; IQR: interquartile range.

Table 2 shows the crude and adjusted analyses for the association between alcohol consumption and depression and anxiety symptoms during the pandemic. In an adjusted analysis, compared to mothers who never drink, those who increased their alcohol consumption showed an increase in the IRR in the depression score of 1.58 times (95%CI 1.25–1.99), and an increased IRR in the anxiety score of 1.66 times (95%CI 1.28–2.15) (Table 2). Table 3 shows the interaction test between alcohol consumption during the pandemic and social isolation. The results showed a consistent association between increased alcohol consumption and depression and anxiety among mothers who reported staying at home for ≥5 days in the last week (IRR 1.82; 95%CI 1.27–2.60, and IRR 1.84; 95%CI 1.21–2.78, respectively) — although formal p-value tests of the effect-modifying role of social isolation were not statistically associated (Table 3).

**Table 2:** Crude and adjusted negative binomial regression model results for the association between alcohol consumption as exposure and maternal mental health. 2019 Rio Grande (RS), Brazil, Birth Cohort.

Alcohol consumption	Depression				Anxiety			
	IRR_crude_ (95%CI)	p-value	IRR_adjusted_ (95%CI)	p-value	IRR_crude_ (95%CI)	p-value	IRR_adjusted_ (95%CI)	p-value
Never drank	Ref.		Ref.		Ref.		Ref.	
Decreased	1.12 (0.87–1.45)	0.378	1.11 (0.86–1.44)	0.423	1.16 (0.87–1.54)	0.316	1.12 (0.84–1.50)	0.426
Not changed	1.08 (0.93–1.26)	0.333	1.09 (0.93–1.27)	0.294	1.09 (0.92–1.29)	0.331	1.09 (0.92–1.30)	0.308
Increased	1.58 (1.25–2.00)	<0.001	1.58 (1.25–1.99)	<0.001	1.67 (1.28–2.17)	<0.001	1.66 (1.28–2.15)	<0.001

95%CI: 95% confidence interval

Adjusted by skin color, living with or without a partner, maternal education, and family income.

**Table 3:** Moderator effects for the association between alcohol consumption as exposure and maternal mental health. 2019 Rio Grande (RS), Brazil, Birth Cohort.

Exposure/Effect modifier		Depression		Anxiety	
		IRR (95%CI)	p-value*	IRR (95%CI)	p-value*
Decreased						
	5 days or more	1.26 (0.86–1.85)	Ref.	1.18 (0.75–1.85)	Ref.
	4 days or less	1.00 (0.71–1.41)	0.376	1.10 (0.76–1.57)	0.796
Not changed						
	5 days or more	1.19 (0.97–1.46)	Ref.	1.20 (0.95–1.53)	Ref.
	4 days or less	0.95 (0.76–1.20)	0.158	0.95 (0.75–1.22)	0.177
Increased						
	5 days or more	1.82 (1.27–2.60)	Ref.	1.84 (1.21–2.78)	Ref.
	4 days or less	1.39 (1.01–1.90)	0.265	1.50 (1.07–2.09)	0.447

IRR: incidence rate ratio.

As moderators, we use the days in a week when people stay at home during the pandemic.

* Interaction test. Global interaction to depression p-value 0.342 and to anxiety p-value 0.534.

## DISCUSSION

In the current study, we observed an increase in both depression and anxiety symptom scores in mothers who reported increased alcohol consumption during the pandemic. Although there was no evidence of a statistically significant effect modification, results were consistent in showing that effect measures of the relationship between alcohol consumption and increased mental health scores were larger among those mothers who remained at home for more extended periods.

It is known that abusive alcohol consumption can be a risk factor for psychiatric disorders, and mothers in the postpartum period are at a higher risk of psychiatric disorders^4,5,7-9^. Furthermore, it has been shown that the COVID-19 pandemic has increased both social isolation and alcohol consumption^15,16^. Our study identified an increase of 5% in alcohol consumption among the interviewed mothers. Previous population-based studies have shown that the pandemic affected the consumption of alcohol, with a consequent impact on mental health. A survey including 1,491 Australian adults was conducted while the country was enforcing social distancing and lockdown measures, showing that those whose alcohol consumption increased were 7 and 8% more likely to have depression and anxiety, respectively^27^. This is in line with what we found in our sample, where mothers whose alcohol consumption increased scored higher on both the depression and anxiety scores. The odds of having a higher score on the depression and anxiety scale are close to 60% higher in mothers who increase their alcohol consumption. The discussion of this relationship is poorly described in the literature. Depression has been attributed to two main mechanisms: neurophysiological and metabolic relationships produced by alcohol consumption that increase the risk of depressive disorder, or to the effects of alcohol on social life and family disruption^28,29^. In addition, the literature suggests that alcohol use is correlated with increased anxiety due to physiological mechanisms^30^.

Regarding the impact of social isolation on alcohol consumption and mental health, a survey of 1,008 young adults residing in the United States found that there was a significant association between loneliness and depression, anxiety, and alcohol use disorder^3^. A Brazilian study with individuals in self-isolation showed 40% higher odds of having anxiety in those who consumed alcohol^31^. We investigated the influence of social isolation on the association between alcohol and mental disorders; however, the number of days the mother stayed at home in the week before the survey did not modify this association. Among the possibilities for the lack of effect of social isolation upon this association is the fact that the number of days the mother stayed at home in the week before the survey may not be a reliable indicator of social isolation, as this variable does not differentiate the reasons for leaving home, time spent outside during the pandemic, and level of social interaction. Besides, the subjective perception of isolation as problematic may be more important than objective measurements. It may be even harder to quantify, as it may depend on variables such as the mother's personality, her social life before the pandemic, and her social interaction level within her residence. Future research should consider multidimensional assessments of social isolation, including subjective feelings of loneliness, quality of social interactions, and household composition, as these factors may more accurately capture the potential moderating role of isolation on the relationship between alcohol consumption and maternal mental health.

In addition, maternal alcohol consumption can negatively impact mother-child interactions and the future of children. There is evidence in the literature that mothers who abuse alcohol are more likely to have children with the same disorder^32^. Another relevant point is maternal mental health, which is often compromised during pregnancy and the first years after birth^33,34^. Depression and anxiety are common diseases during motherhood and can damage not only the child's development but also the relationship with other individuals in the family^35,36^. These disorders may be related to divorce, mother-child deficient relationships, the emergence of psychiatric illnesses throughout life, and a greater chance of suicide^36^. These issues justify the importance of studies like this one in the population, especially among mothers of young children.

In our study, we achieved a 55% response rate compared to the original cohort, with mothers who were wealthier and better educated being the most responsive; however, most online studies have an approximate response rate of 50%^37,38^. It is possible that mental health outcomes were underestimated since it is widely recognised that mental disorders are more prevalent in women with lower income and less education^39^. However, there were no significant changes in terms of depression and anxiety when comparing the baseline and follow-ups^33,34^. In addition, due to the cross-sectional design, it is not possible to infer causality. This is important because the literature points out that there is a bidirectional association between alcohol consumption and mental health problems. In addition, both alcohol consumption and social isolation measurement may have introduced non-differential misclassification, as they were assessed using self-reported measures that do not capture intensity or context. For instance, individuals reporting an increase in alcohol use may have experienced very different levels of consumption, and days spent at home do not account for whether participants lived alone or with others, or for what purpose they left home. These limitations could have biased the associations toward the null or obscured differential effects across subgroups (e.g., individuals living alone *vs.* those living with family). Future research should prioritise more detailed, multidimensional measures to improve accuracy and detect potentially heterogeneous effects, as well as employ longitudinal designs to clarify the directionality of this association. Although both EPDS and GAD-7 have validated clinical cut-off points commonly used for screening purposes, we opted to use the continuous form of these scales to preserve statistical power and capture the full range of symptom severity^40,41^. Moreover, the Minimal Clinically Important Difference (MCID) thresholds are not well established for these instruments in this population^41^, which further supports a continuous approach rather than relying on dichotomised outcomes that may obscure more subtle variations in mental health symptoms. On the other hand, it is essential to note the scarcity of studies on the highlighted issues in Brazil and Latin America, which makes this one of the most extensive follow-up studies available. Recent studies conducted in Latin America and other regions post-pandemic have shown increases in alcohol consumption and worsening maternal mental health outcomes^42^. These findings are consistent with our results and highlight the need for regional longitudinal data to better understand trajectories over time. Another limitation is that mothers of non-live births were not included in the original 2019 Rio Grande Birth Cohort and, consequently, were not eligible for the WebCovid-19 follow-up. Therefore, our findings may not fully capture the experiences and mental health outcomes of women who faced stillbirths or late fetal losses, which are known to be associated with elevated psychological distress^43^. Additionally, participants included in the analysis tended to have higher socioeconomic status and spent more days at home during the pandemic compared to those not included. These differences may have led to lower observed levels of mental health symptoms. They could have influenced the associations between changes in alcohol consumption, time spent at home, and mental health outcomes. Consequently, the effects observed in our study may underestimate the actual burden and the strength of associations in more socioeconomically vulnerable mothers. Despite these limitations, the high baseline response rate (97.8%) supports the internal validity of the study among eligible participants. However, generalizability remains limited to women with live births and to relatively advantaged subgroups.

Finally, our findings indicate a clear association between alcohol consumption and mental health in postpartum women. Although evidence for a moderating role of social isolation was limited, our results underscore the need for further investigation of how isolation may influence this relationship. These findings highlight the importance of targeted interventions for postpartum women, particularly those at risk of increased alcohol consumption or experiencing social isolation. Public health programs should combine mental health support with routine alcohol use screening, implementing culturally sensitive strategies tailored to the Latin American context. Strengthening mental health screening for postpartum women — with a focus on alcohol consumption, anxiety, and depression — is essential. Additionally, accessible interventions, such as telehealth support, may help mitigate the impact of social isolation and provide timely care for mothers in need.
