# Supplementary material for: Alcohol consumption, mental health, and the moderating role of social isolation during the COVID-19 pandemic in southern Brazil
Source: Rev Bras Epidemiol. 2026 Jan 30;29:e260002. doi: 10.1590/1980-549720260002 (PMC12858086; doi:10.1590/1980-549720260002)
Supplement: Supplementary Table 1 [file 1980-5497-rbepid-29-e260002-Suppl01.docx]

**Supplementary Table 1. Sample comparison description between those individuals included and not included in the current analysis. 2019 Rio Grande (Brazil) Birth Cohort.**

| **Variables** | **Not included**  **(n=1,271)** | | | **Included**  **(n=781)** | |
| --- | --- | --- | --- | --- | --- |
|  | **n** | **% (CI95%)** | | **n** | **% (CI95%)** |
| **Maternal self-reported skin color** | | |  |  |  |
| Black | 125 | 9.8 (8.3;11.6) | | 49 | 6.3 (4.8;8.2) |
| Brown | 195 | 15.3 (13.5;17.4) | | 107 | 13.7 (11.4;16.3) |
| White | 951 | 74.8 (72.4;77.1) | | 625 | 80.0 (77.1;82.7) |
| **Maternal schooling** |  |  | |  |  |
| Elementary school | 444 | 34.9 (32.4;37.6) | | 163 | 20.9 (18.1; 23.9) |
| High school | 608 | 47.8 (45.1;50.6) | | 370 | 47.4 (43.9; 50.9) |
| College or more | 219 | 17.2 (15.2;19.4) | | 248 | 31.7 (28.6; 35.1) |
| **Number of people in the household** | |  | |  |  |
| <3 | 742 | 58.4 (55.6;61.1) | | 520 | 66.6 (63.2; 69.8) |
| 3 or 4 | 275 | 21.6 (19.5;24.0) | | 147 | 18.8 (16.2;21.7) |
| 5 or more | 254 | 20.0 (17.9;22.3) | | 114 | 14.6 (12.3;17.3) |
| **Family income**^€^ |  |  | |  |  |
| 1^st^ (poorest) | 497 | 40.1 (37.4;42.9) | | 219 | 28.6 (25.5; 31.9) |
| 2^nd^ | 374 | 30.2 (27.7;32.8) | | 223 | 29.1 (26.0;32.5) |
| 3^rd^ (richest) | 367 | 29.6 (27.2;32.3) | | 323 | 42.2 (38.7; 45.8) |
| **Days in a week spent at home during the pandemic** | | | |  |  |
| 5 days or more | 111 | 42.5 (36.6–48.6) | | 447 | 57.3 (53.8–60.7) |
| 4 days or less | 150 | 57.5 (51.4–63.4) | | 333 | 42.7 (39.3–46.2) |
| **Alcohol consumption during the pandemic** | | | |  |  |
| Never drank | 182 | 70.5 (64.7–75.8) | | 572 | 73.3 (70.1–76.3) |
| Decreased | 17 | 6.6 (4.1–10.4) | | 44 | 5.6 (4.2–7.5) |
| Not changed | 47 | 18.2 (13.9–23.4) | | 125 | 16.0 (13.6–18.8) |
| Increased | 12 | 4.7 (2.7–8.0) | | 39 | 5.0 (3.7–6.8) |
|  | **n** | **Median (IQR)** | | **n** | **Median (IQR)** |
| **Depression score** | - | - | | 781 | 8.0 (3–13) |
| **Anxiety score** | - | - | | 781 | 7.1 (5.6) |

95%CI: 95% confidence interval; IQR: interquartile range

Note: The N sample from those not included in the current analysis also included the inclusion criteria for the WebCovid-19 study (i.e., we did not consider the total baseline cohort for counting the difference).
